# A Case of Severe Drug-induced Liver Injury Caused by Over the Counter Herb (Cinnamon): Review of Literature

**DOI:** 10.5005/jp-journals-10018-1284

**Published:** 2019-02-01

**Authors:** Hiromi Higaki, Morikazu Onji, Satoshi Takeji, Takahide Uehara, Keitaro Kawasaki, Youhei Kashimoto, Takatoshi Murakami, Tomotaka Yamaguchi, Jiro Miyaike, Masaki Oomoto, Masanori Abe

**Affiliations:** 1 Center of Postgraduate Medical Education, Saiseikailmabari Hospital, Imabari, Japan; 2 Department of Internal Medicine and Gastroenterology, SaiseikaiImabari Hospital, Imabari, Japan; 3 Department of Gastroenterology and Metabology, Ehime University Graduate School of Medicine, Toon, Japan

**Keywords:** Cinnamon, Drug-induced liver injury, Herb medicine, Over-the-counter, Severe hepatitis.

## Abstract

A case of severe drug-induced liver injury caused by over the counter (OTC) herb medicine, is reported here. A 40-year-old male took herb drugs “Za ga-doKowa®,” “Ohta-Isan®.” These two drugs contained the same two herb medicines (cinnamon, fennel). About 4 months later after taking medicine, jaundice appeared. Prothrombin time activation (PT) was 45%, aspartate transaminase (AST) was 1104 IU/l, and total bilirubin (T-bil) 14.7 mg/dL. Serum tests for hepatitis viruses (A, B, C, E) were negative. Lymphocyte stimulating test was positive for Za ga-do Kowa ® and Ohta-I Isan®. Liver 3D constructed by construct-CT revealed findings of the potato-like liver. The liver biopsy specimen revealed multilobular hepatic necrosis accompanied by scar formation, severe zonal degeneration and necrosis of hepatocytes mainly in the central area of the lobule.

In the reported 13 cases of cinnamon-induced liver diseases, there has been a severe abnormality of PT and T-bil. Biopsy findings of these cases showed wide ranges of necrosis. Liver injury due to cinnamon shows very severe damages, and the possibility of liver failure due to cinnamon may be imminent.

**How to cite this article:** Higaki H, Onji M, Takeji S, Uehara T, Kawasaki K, Kashimoto Y, Murakami T, Yamaguchi T, Miyaike J, Oomoto M, Abe M. A Case of Severe Drug-induced Liver Injury Caused by Over the Counter Herb (Cinnamon): Review of Literature. Euroasian J Hepatogastroenterol, 2018;8(2):167-171.

## INTRODUCTION

Herb medicines have been attaining considerable popularity in Japan because of their so-called safety and widespread availability. Several drugs of herbal origin are now available as OTC drugs. However, increasing evidence have been piled up about their adverse effects. Also, it is known that the ratios of liver injury and adverse effects are very high with the OTC herb medicine than legitimate medical herb medicine.^1^

Here, we report a case of severe drug-induced liver injury (DILI) caused by OTC herb medicine (Za ga-do Kowa® and Ohta-Isan®). These drugs are available commercially at almost all places of Japan. Finally, we have accomplished a literature review with cases like the reported one.

## CASE REPORT

The patient that has been reported here was a 40-year-old male. Chief complains were an epigastric pain, abdominal bloating in postprandial time, and progressive increase of darkish color of his urine. The previous history of illness was not contributory to his present illness. Family history revealed that his uncle had colon cancer and lung cancer, however, a family history of hepatitis or jaundice or liver diseases could not be substantiated. The patient was a smoker and has been consuming about 10 cigarettes per day for the last 24 years. He was a social drinker and consuming about 350 mL of beer once a week. The patient reported no history of allergy. Also, there was no history of taking nutrient supplements. He has been working in recycling industry. There was no history of previous surgery or blood transfusion.

According to the history of present illness, the patient started taking oral Za ga-do Kowa® from January 2016 for his constipation. He began to feel heartburn from around March 2016 and then started to consume Ohta-Isan® and Gasuto-ru®. From early April 2016, he frequently felt malaise as well as epigastric pain. Around mid-April 2016, he noticed yellowish skin. From May 2016, the color of urine was found to be dark and brown. The stool color became somewhat whitish.

Along with time and mainly from mid-May 2016, the extents of malaise feelings became exacerbated. On May 17th, 2016, he noticed considerable nausea, noticeable loss of appetite, and increasing order of malaise. On May 19th, 2016, he was admitted to a clinic for his complaints. The local clinic referred him to our department on that day, and he was admitted to Imabari Saiseikai Hospital, Ehime, Japan.

On admission, physical examination revealed that his height was 168.7 cm and weight was 84.6 kg. He was conscious of admission. The skin and bulbar conjunctiva were icteric. However, there was no murmur at heart or lung. There were marks of insect bites on extremities. Also, acne was found on the back. Bowel sounds were normal. The abdomen was flat, soft and without any noticeable tenderness. Liver and spleen were not palpable. He had a blood pressure of 116/72 mm of Hg. Pulse rate was within normal range (88 beats per minute).

The laboratory findings on admission have been shown in Table 1 and described below: aspartate trans-aminase (AST); 988 IU/I, alanine aminotransferase (ALT); 847 IU/I, alkaline phosphatase (ALP); 320 IU/I, gamma-glutamyl transpeptidase (-GTP); 126 IU/I, total bilirubin (T-bil); 11.88 mg/dl, direct bilirubin (D-bil); 7.77 mg/dl, PT-activation; 58%, lactate dehydrogenase (LDH); 386 IU/I, alpha fetoprotein (AFP); 73.9 ng/mL, carcinoembry-onic antigen (CEA); 2.8 ng/mL and PIVKA2; 30mAU/mL. The patient was negative for markers of acute infections (IgM type antibodies) against cytomegalovirus (CMV), herpes simplex virus (HSV), Epstein-Barr virus (EBV), hepatitis A virus (HAV), hepatitis B virus (HBV), hepatitis C virus (HCV) and hepatitis E virus (HEV). The titers of anti-mitochondrial antibody titer [<20 (-ve)], anti-smooth muscle antibody (<20) and anti-LKM-1 antibody were negative. Serum mac-2 binding protein glycosylation isomer (M2BPGi) was 9.21 and serum hepatocyte growth factor (HGF) was 2.31 ng/mL.

**Table 1:** **Table 1:** Laboratory data on admission

*During Admission*							
WBC		6300/μL		NH4		90 μg/mL	
RBC		4400000/μL		AFP		73.9 ng/mL	
Hemoglobulin		13.6 g/dL		CEA		2.8 ng/mL	
PLT		209000/μL		CA19-9		109/8U/mL	
Atypical Ly		0.5%					
ALT		847 IU/L					
AST		988 IU/L					
LDH		392 IU/L					
ALP		320 IU/L					
T-GTP		126 IU/L					
CRP		0.73 mg/L					
ESR		2-8 mm/hour					
Ferritin		472.8 /mL					
Fe		260 μg/mL					
Ceruloplasmin		30.3 mg/dL					
CHE		216 IU/L					
TG		145 mg/dL					
HDL-C		11 mg/dL					
LDL-C		94 mg/dL					
Albumin		3 gm/dL					

WBC, White blood count; RBC, Red blood corpuscle; PLT, Platelet; ALT, Alanine aminotransferase; AST, Aspartate aminotransferase; LDH, lactate dehydrogenase; ALP. Alkaline phosphatase; T-GTP; Glutamyl transpeptidase; CRP, C-reactive protein; ESR, Erythrocyte sedimentation rate; Fe, Iron; CHE, Cholinesterase; TG, Triglycerides; HDL-C, high-density lipoprotein cholesterol; LDL-C, high-density lipoprotein cholesterol; NH4, ammonia; AFP, Alpha feto protein; CEA, Carcinoembryonic antigen

Liver elastography showed VTQ: 4.0 m/s (3 times of average). This hardness corresponds to Inuyama classification F4 or more. (VTQ Reference standard value: F0 0.67 to 1.44 m/s, F1 0.81 to 1.63 m/s, F2 0.89 to 1.85m/s, F3 1.11 to 2.29m/s, F4 1.52 to 2.94m/s Multicenter 2012)). Fibro scan showed liver stiffness of 7.5kPa (just above the upper limit of normal).

**Fig. 1:**
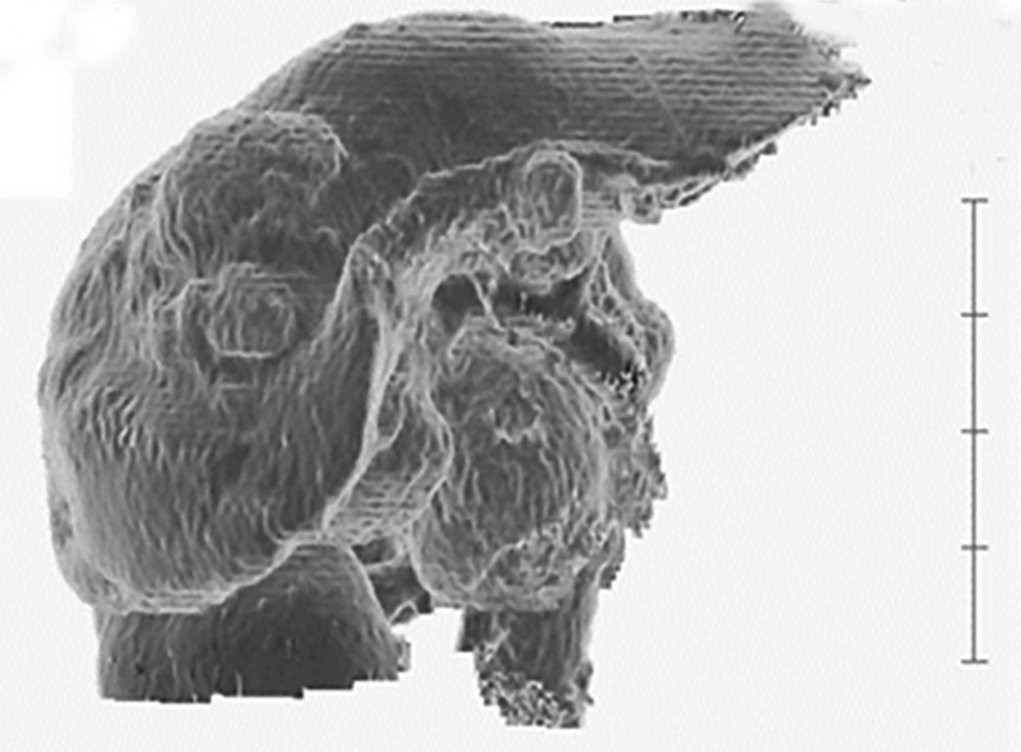
Volume rendering imaging of the liver showing a potato liver. It is the liver on the 51st day after hospitalization. Volume rendering image (Liver 3D construction image by Vincent) revealed the irregular atrophy at the liver interior ~ anterior compartment and potato-like regrowth

Contrast CT showed the irregular atrophy of the interior ~ anterior compartment of the liver with features of fatty liver. Obvious space occupying lesion (SOL) could not be visualized in the liver. Volume rendering image of the liver revealed potato-like regrowth (potato-liver) (Fig. 1). Measurement of liver volume showed that it was 1428 mL in May 2016, 1007 mL in July 2016 and 1259 mL in November 2016. Along with the improvement of liver function, recovery of volume and augmentation of the anterior zone were seen.

**Figs 2A to C:**
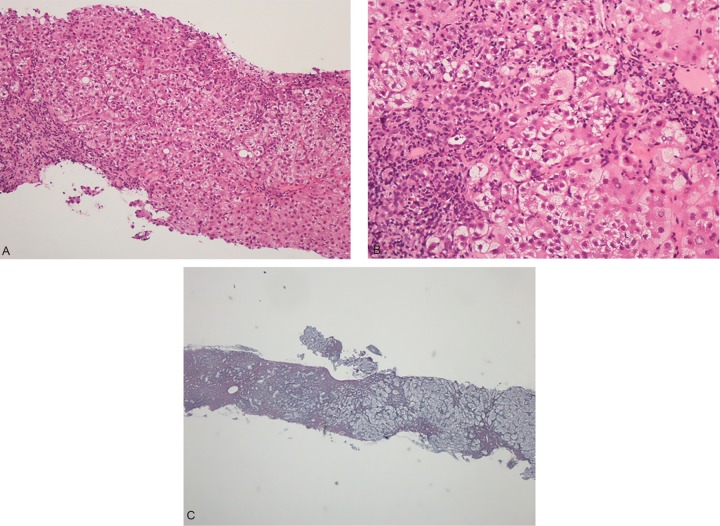
(A) Liver biopsy specimen showing inflammatory cell infiltration withy relatively high proportions of lymphocytes (small number of eosinophils and neutrophils); (B and C) There were partially bridging necrosis and fibrosis in liver bJiopsy specimen.

The liver biopsy specimen revealed features of liver cell necrosis (multilobular hepatic necrosis) and infiltration of inflammatory cells containing relatively higher ratios of lymphocyte accumulation (Fig. 2A). Partially bridging fibrosis, hyperplasia of cholangiole, and expansion of fibrous portal areas was also shown (Figs 2B and C). Vacuolation of the nucleus of liver cells (glycogen nucleus) were few and fatty depositions was negligible. Features of cholestasis and iron deposition could not be marked. The findings of liver biopsy were compatible with a diagnosis of DILI.

As all serum viral markers including hepatitis viruses (A, B, C, and E) were negative, we performed the drug lymphocyte stimulation test (DLST). DLST for Za ga-do Kowa® and Ohta-Isan® were positive, however, that for Gaster® was negative. Cinnamon and fennel are components common to Za ga-do Kowa® and Ohta-Isan®, and they showed borderline stimulation (Table 2). Drug-induced liver injury score (DDW-J 2004) was four points.^2^

After admission, the patient stopped taking medicine. Subjective symptoms gradually disappeared. However, it was the sixth week after hospitalization, the liver enzyme rises and bilirubin re-elevation started. At one time PT became 45% and T-bil increased to 14.8mg/dL.

It presented a severe liver injury. After that, we were given ursodeoxycholic acid and late evening snack (LES) therapy. We continue treating in a protective way for liver, and the patient left the hospital after 15th week when the levels ALT and AST came below 100 IU/L, and the levels of serum bilirubin decreased to 4 mg/dL.

**Table 2:** **Table 2:** Drug-induced Lymphocyte Stimulation Test

*Za ga-doKowa ® Sceichojo PC*		*Drug*		*Ohta-Isan*			
Simulation		1.8		Simulation		1.9	
index				index			
Maximum		271 CPM		Maximum		399 CPM	
reaction level				reaction level			
Free Culture		154 CPM		Free Culture		211 CPM	
PHA Culture		4673		PHA Culture		98041	
Cinnamon				Fennel			
Simulation		1.4		Simulation		1.5	
index				index			
Maximum		296 CPM		Maximum		322 CPM	
reaction level				reaction level			
PHA control		216 CPM		Free Culture		216 CPM	
PHA Culture		172028		PHA Culture		172028 CPM	

PHA, phytohemagglutinin; CPM, count per minute

**Table 3:** **Table 3:** Review of literature

*Drug*		*Age*		*Sex*		*Total bilirubin*		*Alanine aminotransferase*		*Alkaline phosphatase*		*Reference*	
Kinsigan		51		F		1.5		634				5	
Kinsigan		46		F		0.8		207		129		5	
Kinsigan		59		F		1.3		829				6	
Kinsigan		62		M		17.7		2002		294		7	
Kinsigan		27		F		28.5		166				8	
Kinsigan		66		F		0.5		358		356		9	
Kinsigan		54		F		38.3		822		256		10	
Saireitou		57		F		8.5		1077		311		11	
Shikokeisikankyoutou		47		F		4.3		1112		356		12	
Shikokeisikankyoutou		42		F		2		1167		686		13	
Kaigen		66		M		14.7		1104		399		14	

## DISCUSSION

The study presented here provides strong evidence that Za ga-do Kowa® and Ohta-Isan® might be responsible for liver injuries of this patient. First, the patient has been consuming these two drugs, and he was negative for the relevant markers of viruses that may cause acute hepatitis. The next, cessation of these drugs resulted in improvement of liver functions and the patient ultimately went back home without any specific therapy. Also, the patient showed positivity of DLST to these two herbal drugs. Taken together, this is a case of DILI possibly caused by two OTC herb drugs.

The common components of these two herb drugs are cinnamon and fennel. Ten mg of cinnamon and fennel is included in one dose of Za ga-doKowa®. On the other hand, 90 mg of cinnamon and 24 mg of fennel are included per one dose of Ohta-Isan®. In this patient, liver damage progressed by adding Ohta-Isan®. DLST showed borderline positivity to cinnamon and fennel. The possibility of the false negativity of DLST is a reality, and it is difficult to assess the real impact of DLST by one testing.^2-4^ However, the case history strongly indicates a role of these components in inducing DILI in this patient. There is a lack of information about fennel-induced DILI. However, there are some reports of DILI caused by cinnamon.

The liver injury caused by cinnamon has been reported previously (searched via igaku-chuo-zasshi),^5-15^ (Journal in Japanese). The history and details of the present patient are compatible with the reported patients in the literature (Table 3). Cinnamon usually induces severe liver injury (high level of serum total bilirubin, low levels of prothrombin time, multilobular hepatic necrosis). It has also been shown that coumarin in cinnamon has the hepatotoxicity, and a warning is given to the excessive intake with the food supplement in Germany from 2006.^16^

As of today, the commercial market of Chinese herbal medicine is about around 141 billion yen and this represents about 2% of market sizes of the whole medical supplies. The use of Chinese medicine is on a growing trend compared to other drugs (37% *vs.* 7%) in the last 6 years (2006-2012).^17,18^ Under these realities, there must be more works about the adverse effects of herbal drugs.

Also, in Relief System for Sufferers from Adverse Drug Reactions, the hepatobiliary disorder happened with Chinese medicine is placed next to a central nervous system medicine. And approximately 30% was caused by the usage of Chinese medicine available in the OTC.^19^ Also, liver injury represents the major side effects of OTC drugs (about 41.4%).^1^

In conclusion, we presented a case of severe hepatitis due to the cinnamon from herb medicine of OTC drug. Liver injury due to cinnamon shows a very severe type of DILI. Immediate and emergency public health measures and health different health education measures should be adopted to make rationale usage of OTC-based Chinese herbal drugs.

## References

[1] Ito T “Examination of Frequency Nature of Side Effects Caused by Over-The-Counter Kampo Formulations Based on the Date Published by the Japanese Ministry of Health, Labour and Welfare.” Kampo medicine 67.2 (2016).

[2] Takikawa H, Onji M, Takamori Y (2005). A proposal of the diagnostic scale of drug drug-induced hepatic injury. Heptol Res.

[3] Sugihara T, Koda M, Okamoto T (2016). The usefulness of second drug-induced lymphocyte stimulation tests (DLST). Kanzo 57.11.

[4] Onji M, Yamashita Y, Ohta Y (1981). The immunodiagnosis of drug-induced allergic hepatitis, by macrophage activating factor assay. Jpn,L Clin. Immunol.

[5] Sato E, Maeta H, Honda K, Ito T, Tsukioka S, Shibasaki K (1984). A case report of herb medicine induced hepatic injury. Acta Hepatol Jap.

[6] Tazawa J, Mae S, Sakai H, Nishimura M, Hasumura Y, Takeu-chi J (1985). A case of herb drug-induced liver injury showing the different morphological findings during the disease course of two years”. Kanzo 26.12.

[7] Yamazaki K, Suzuki K, Sato K, Ouchi K, Yoshinari H, Isozaki I (1991). Herbal drug-induced fulminant hepatitis. Kanzo 32.7.

[8] Mizoguchi Y, Miyajima K, Sakacami Y, Yamamoto S (1986). A serious case of drug-induced allergic hepatitis by a herbal medicine. The Journal of the Japanese Society of Internal Medicine 75.10.

[9] Maeta H, Shibuya T, Sato I (1991). “ A case of drug-induced allergic hepatitis by Kinshigan”. The Japanese Journal of Clinical and Experimental Medicine 68.

[10] Ikeda R, Kanaoka M, Fujisawa T, Doi Y, Kumamoto I, Onji M (1994). A case of drug induced liver injury caused by Kinshigan”. Gastroenterological Endoscopy 36.7.

[11] Nakada T, Kawai B (1996). “A case of hepatic injury induced by Sai-rei-to”. Kanzo 37.4.

[12] Kobayashi F, Nakamura H, Numata M, Wasada M, Shiraishi K, Itakura M (1997). A case of drug-induced liver injury caused by Saiko-Keishi-Kankyoto with thrombocytopenia. Journal of Japanese Society of Gastroenterology. 94.10.

[13] Hosonuma K, Yuasa K (2003). “A case of reproducible hepatic injury induced by three kinds of herbal medicine”. Kanzo 44.1.

[14] Mizoguchi Y, Yoshiyasu K, Tsutsui H, Myyajima K, Sakagami Y, Tojo T (1985). A case of drug-induced allergic hepatitis by herbal medicine”. Kanzo 26.3.

[15] Kamigaki M, Nakazawa I (2001). “ a case of drug-induced liver injury due to Kakkon-o”. Internal Medicine 87.

[16] Bundesinstitu fur Risikobewertung (BfR). http://www.bfr.bund.de/cm/343/hohe_taegliche_aufnahmemengen_von_zimt_gesundheitsrisiko_kann_nicht_ausgeschlos-sen_werden.pdf.

[17] Ministry of Health, Labour and Welfare ,Pharmaceutical industrial production dynamics annual report, 2012, Table 2. <http://www.mhlw.go.jp/file/04-Houdouhappyou-10807000-Iseikyoku-Keizaika/0000069199.pdf>.

[18] Fujitu research institute -Fine-view about the industry of the oriental medicine(1) domestic crude drug and quality evaluation technology-. <http://www.fujitsu.com/jp/group/fri/column/opinion/201501/2015-15.html>.

[19] Yoshitake M, Asano K (2016). “About the relief system of the hepatobiliary disorder in the relief system for sufferers from adverse drug reactions”. Kanzo 57.3.

